# Seroepidemiology and Risk Factors of *Toxoplasma gondii* Infection among the Newly Enrolled Undergraduates and Postgraduate Students in China

**DOI:** 10.3389/fmicb.2017.02092

**Published:** 2017-10-26

**Authors:** Na Yang, Dawei Wang, Mengen Xing, Chenghuan Li, Jiaqi Li, Anhe Wu, Xiaoyu Sang, Ying Feng, Ning Jiang, Qijun Chen

**Affiliations:** Key Laboratory of Zoonosis of Liaoning Province, College of Animal Science and Veterinary Medicine, Shenyang Agricultural University, Shenyang, China

**Keywords:** *Toxoplasma gondii*, seroepidemiology, risk factors, students, China

## Abstract

*Toxoplasma gondii* is an obligate intracellular zoonotic parasite, infecting warm-blood animals including humans. Previous serological surveys of *T. gondii* infection have focused on people of different occupations and special groups, such as slaughterhouse workers, AIDS patients and pregnant women. To investigate the potential impact of *T. gondii* infection on the health of young students, the prevalence of *T. gondii* infection and associated risk factors among the newly enrolled undergraduates and postgraduate students were investigated. A total of 3,569 newly enrolled students (age range: 15- to 37-years-old, median 26 years) from various regions of China were recruited in this study. The serum samples were tested for the presence of *T. gondii* specific IgG by the modified agglutination test (MAT). Questionnaires were used to collect information on risk factors for *T. gondii* infection. Sixty-five (1.82%) out of 3,569 participants were seropositive for IgG antibodies to *T. gondii* by MAT (titer≥1:20). Four variables were found to be positively associated with *T. gondii* infection, including primary geographical location, living in rural areas, gardening or agriculture, and drinking unboiled water by the univariate logistic regression, and only gardening or agriculture was the independent risk factor for *T. gondii* positivity by using multivariate logistic regression in this study, which may provide information to guide future research and control policies.

## Introduction

*Toxoplasma gondii* is an obligate intracellular zoonotic parasite, infecting warm-blood animals, including humans. It has been reported that one-third of the human population worldwide and more than 7% of the population in China are chronically infected with *T. gondii* (Dubey, 2010; Zhou et al., 2011; Qin et al., 2014). Humans can be infected through three major ways including consumption of undercooked meat containing *T. gondii* tissue cysts, ingesting oocysts-contaminated water, soil, vegetables and fruits, and transmission from mother to fetus during pregnancy (Dubey, 2010; Wang et al., 2016).

In women, primary infection during pregnancy can cause severe damage to fetus and newborns including stillbirth, abortion and blindness. It can cause severe infections in individuals with compromised immune systems such as patients with AIDS, cancer treatment or organ transplantation (Dubey, 2010; Zhou et al., 2011). Immune competent individuals infected with *T. gondii* are generally asymptomatic; however, *T. gondii* infection has been associated with neuropsychiatric disorders suggesting that latent infection may have subtle neurological effects (John et al., 2015).

Toxoplasmosis has become a notifiable disease in China since the first epidemic survey on *T. gondii* infection in humans was carried out in Guangxi Province in 1978 (Chen et al., 2005). Previous serological surveys of *T. gondii* infection have focused on people of different occupations, such as slaughterhouse workers, dairy workers, veterinarians, meat-processing workers, cook, and animal breeder, and special groups, such as pregnant women, AIDS and cancer patients, and intravenous drug users (IVDU) in China (Zhou et al., 2011). However, the infection of *T. gondii* in young students and its potential impact has not been investigated. In this study, the prevalence of *T. gondii* infection and associated risk factors among the newly enrolled 3,569 undergraduates and postgraduate students of Shenyang Agricultural University from all over China was investigated.

## Materials and Methods

### Participants

A total of 3,569 whole blood samples of newly enrolled undergraduates and postgraduate students of Shenyang Agricultural University originated from various regions of China were collected in September 2016 to survey the presence of *T. gondii* specific antibodies. The-original regions of the students are mainly located in eastern, southern, central, north, northwest, southwestern, and northeastern regions of China. The age of the newly enrolled students ranged from 15 to 37 years (median 26 years).

### Ethics Statement

This study was carried out in accordance with the recommendations of the Guidelines for Using Subjects from Humans and Animals, Ethical Committee of Shenyang Agricultural University (Clearance number 2015-CAV-01) with written informed consent from all participants. All subjects gave written informed consent in accordance with the Declaration of Helsinki.

### Serological Testing

The sera of all participants were tested for the specific IgG to *T. gondii* by the modified agglutination test (MAT) as described by Dubey and Desmonts (1987) and the *T. gondii* Whole Cell Antigen was purchased from Kerafast, Inc. (Boston, MA, United States). MAT has been regarded as the gold-standard for *T. gondii* infection test. Twofold dilutions of the sera were performed from 1:20 to 1:1,280. The test was regarded positive when a layer of agglutinated parasite antigen was formed in wells at dilutions of 1:20 or higher; positive and negative controls were included in each test. Each serum was tested for three times.

### Questionnaire

The questionnaire contained information of basic demographic data, including age, gender, education background and residence before university. Possible risk factors, including drinking unboiled water, raw or not well-cooked meat (including lamb, beef, pork, fish, oyster) and raw vegetable consumption, animal contacts (cats or dogs), gardening or agricultural activities, blood transfusion and living in urban areas or countryside.

### Statistical Analysis

For the statistical analysis, the SPSS 13.0 software package (IBM, Armonk, NY, United States) was used. *P*-values less than 0.05 were considered statistically significant. Logistic regression was used to analyze the association between *T. gondii* infection and potential risk factors. Multivariate logistic analysis was further performed with the full model, including all potential risk factors in the analyses.

## Results

### Seroprevalence of *T. gondii* Infection

In the present study, 65 (1.82%) of the 3,569 participants were seropositive for anti-*T. gondii* IgG by MAT, with titers of 1:20 in 35, 1:40 in 20, and 1:80 in 10 participants. The seroprevalence of *T. gondii* infection in postgraduate students (2.46%) was higher than that in undergraduates (1.63%), however, the difference was not statistically significant (*p >* 0.05) (**Tables 1, 2**). By gender, the anti-*T. gondii* IgG seropositive rates was 1.91% (26/1360) in men and 1.31% (29/2209) in women (*p* = 0.751), respectively (**Tables 1, 2**).

**Table 1:** Demographic characteristics and seroprevalence of *Toxoplasma gondii* infection in 3,569 participants tested by the modified agglutination test (MAT).

Characteristics		Samples	No. positive	Positive rate (%)
Region	East China	335	5	1.49
	South China	36	2	5.55
	Central China	279	6	2.15
	North China	439	6	1.37
	Northwest China	152	3	1.97
	Southwest china	228	15	6.58
	Northeast China	2,076	28	1.34
	Unknown regions	24	0	0
Gender	Male	1,360	26	1.91
	Female	2,209	29	1.31
Age	16–19	2,362	37	1.57
	20–25	1,122	25	2.22
	26–37	85	3	3.52
Education	Undergraduate	2,756	45	1.63
	Postgraduate	813	20	2.46
Blood group type	A	304	10	3.29
	B	398	4	1.01
	AB	155	1	0.64
	O	437	8	1.83
	Unknown	2,275	42	1.84
Total		3,569	65	1.82


**Table 2:** Univariate analysis of the variables associated with *T. gondii* seroprevalence in participants tested by MAT.

Variable	Blood donor No.	Seropositivity (%)	Odds ratio (95% Confidence internal)	*P*-value
Geographical location				
Unknown	24	0	0.000	0.998
North of the Yangtze River	3,045	1.44	0.4 (0.2–0.9)	0.026^∗^
South of the Yangtze River	303	4.62	1.3 (0.5–3.3)	0.562
Yangtze River Province	197	3.55	1	
Living in rural areas or city				
City	2,183	1.33	0.5 (0.3–0.8)	0.009^∗^
Rural areas	1,386	2.59	1	
Cat in the household				
Yes	299	1.67	0.000	0.998
No	3,270	1.83	1	
Cat in the neighborhood				
Yes	37	0	0.9 (0.4–2.3)	0.841
No	3,532	1.84	1	
Keep a dog				
Yes	773	1.16	0.6 (0.3–1.2)	0.127
No	2,796	2.01	1	
Drink unboiled water				
Yes	1,340	2.46	1.7 (1.1–2.8)	0.028^∗^
No	2,229	1.43	1	
Undercooked beef meat/lamb consumption				
Yes	240	1.25	0.7 (0.2–2.1)	0.496
No	3,329	1.86	1	
Undercooked pork meat consumption				
Yes	46	0	0.000	0.998
No	3,523	1.85	1	
Raw fish consumption				
Yes	585	1.71	0.926 (0.5–1.8)	0.825
No	2,984	1.84	1	
Fresh oyster consumption				
Yes	544	2.21	1.3 (0.7–2.4)	0.467
No	3,025	1.75	1	
Undercooked vegetables consumption				
Yes	2,198	1.55	0.7 (0.4–1.1)	0.123
No	1,371	2.26	1	
Gardening or agriculture				
Yes	644	4.04	3.1 (1.9–5.2)	0.000^∗^
No	2,925	1.33	1	
Blood transfusion				
Yes	41	0	0.000	0.998
No	3,528	1.85	1	
Blood group type				
Unknown	2,275	1.84	2.9 (0.4–21.2)	0.295
O	437	1.83	2.9 (0.4–23.1)	0.322
A	304	3.29	5.2 (0.7–41.3)	0.116
B	398	1.01	1.6 (0.2–14.1)	0.690
AB	155	0.64	1	
Gender				
Male	1,360	1.91	1.1 (0.7–1.8)	0.751
Female	2,209	1.31	1	
Education level				
Undergraduate	2,756	1.63	0.7 (0.4–1.1)	0.124
Postgraduate students	813	2.46	1	


^∗^*Statistically significant*.

**FIGURE 1:**
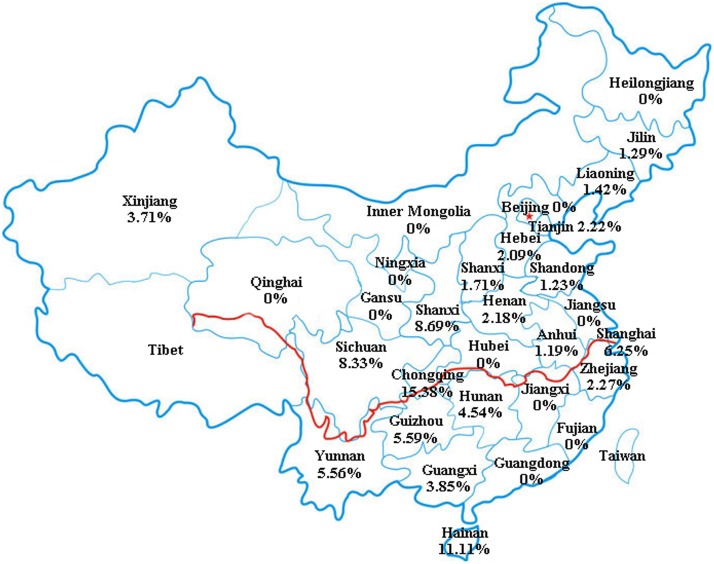
Geographical locations where this study was carried out.

By geographic region, seroprevalence of *T. gondii* infection was 1.49% (5/335) in Eastern China, 5.55% (2/36) in Southern China, 2.15% (6/279) in Central China, 1.37% (6/439) in Northern China, 1.97% (3/152) in Northwest China, 6.58% (15/228) in Southwest China, 1.34% (28/2,076) in Northeast China (**Figure 1** and **Table 1**). In addition, the seroprevalence of *T. gondii* infection varied in different geographical regions, ranging from 1.44 to 4.62%, with a general tendency of a lower prevalence in the North and higher in the South (**Tables 2, 3**). Students originated from provinces of Hainan and Chongqing showed the significantly higher infection rates (11.11 and 15.38%, respectively).

**Table 3:** The seroprevalence of *T. gondii* infection in 3,569 participants from different geographical regions in China.

	Province	Samples	No. positive	Positive rate (%)
East China	Shandong	162	2	1.23
	Jiangsu	17	0	0
	Anhui	84	1	1.19
	Zhejiang	44	1	2.27
	Fujian	12	0	0
	Shanghai	16	1	6.25
South China	Guangdong	1	0	0
	Guangxi	26	1	3.85
	Hainan	9	1	11.11
Central China	Hubei	18	0	0
	Hunan	22	1	4.54
	Henan	229	5	2.18
	Jiangxi	10	0	0
North China	Beijing	12	0	0
	Tianjin	45	1	2.22
	Hebei	143	3	2.09
	Shanxi	117	2	1.71
	Inner Mongolia	122	0	0
Northwest China	Ningxia	17	0	0
	Xinjiang	27	1	3.71
	Qinhai	13	0	0
	Shanxi	23	2	8.69
	Gansu	72	0	0
Southwest China	Sichuan	36	3	8.33
	Yunnan	18	1	5.56
	Guizhou	161	9	5.59
	Chongqing	13	2	15.38
Northeast China	Liaoning	1,901	27	1.42
	Jilin	77	1	1.29
	Heilongjiang	98	0	0
	Unknown regions	24	0	0
	Total	3,569	65	1.82


The participants were also allocated to 3 age groups: 16- to 19-years-old, 20- to 25-years-old, and 26- to 37-years-old. The seroprevalence of *T. gondii* infection varied in different age groups, ranging from 1.57 to 3.52%, with a general tendency for older participants to have a higher prevalence, compared with younger participants (**Table 1**).

### Risk Factors for *T. gondii* Infection

In the univariate analysis, four variables were found to be associated with anti*-T. gondii* IgG positivity, including geographical location, living in rural areas, gardening or agriculture, and drinking unboiled water (**Table 2**). Further analysis using multivariate logistic regression revealed that gardening or agriculture was independent risk factor for *T. gondii* seropositivity (**Table 4**).

**Table 4:** Multivariate logistic regression with full model for risk factors of *T. gondii* infection.

Variable	Odds ratio (95% Confidence internal)	*P*-value
Geographical position (unknown vs. Yangtze River Province)	0.0	0.998
Geographical position (North of the Yangtze River vs. Yangtze River Province)	0.4 (0.2–1.0)	0.050
Geographical position (South of the Yangtze River vs. Yangtze River Province)	1.2 (0.4–3.0)	0.748
Living in rural areas or city	0.8 (0.4–1.6)	0.620
Cat in the household	0.0	0.998
Cat in the neighborhood	0.8 (0.3–2.2)	0.728
Keep a dog	0.4 (0.2–0.9)	0.021
Drinking unboiled water	1.6 (0.9–2.8)	0.084
Undercooked beef meat/lamb consumption	0.9 (0.3–3.2)	0.889
Undercooked pork meat consumption	0.0	0.998
Raw fish consumption	1.1 (0.5–2.6)	0.845
Fresh oyster consumption	2.0 (0.9–4.4)	0.087
Undercooked vegetables consumption	0.7 (0.4–1.2)	0.173
Gardening or agriculture	3.0 (1.5–5.7)	0.001
Blood transfusion	0.0	0.998
Blood group type (Unknown vs. AB)	2.4 (0.3–17.5)	0.403
Blood group type (O vs. AB)	2.8 (0.3–22.9)	0.334
Blood group type (A vs. AB)	4.7 (0.6–37.8)	0.143
Blood group type (B vs. AB)	1.5 (0.2–13.3)	0.740
Gender	0.8 (0.5–1.4)	0.522
Education level	0.6 (0.3–1.0)	0.058


## Discussion

In this study, *T. gondii* infection among newly enrolled undergraduates and postgraduate students were systematically investigated. The overall prevalence of *T. gondii* antibodies among these participants was 1.82% (65/3,569); the infection rates of the students coming from eastern, southern, central, north, northwest, southwestern and northeastern regions of China are 1.49, 5.55, 2.15, 1.37, 1.97, 6.58, and 1.34%, respectively. These results indicated that *T. gondii* infection occurred earlier in students in all geographical regions in China, but at lower infection rate than that of total population reported earlier (Xiao et al., 2010; Chiang et al., 2012; Yang et al., 2013). The differences between the current study and the previous reports were likely due to the study population. Studies earlier focused predominantly on adult groups with different professions and disease exposures or co-infection (Zhou et al., 2011). Here, we only focused young students before university. It was reported that the seroprevalence of *T. gondii* infection in students was 3.2% in São Paulo State University (UNESP), in Assis, São Paulo state, Brazil (Rodrigues et al., 2015), which was higher than the infection rate in this study. Moreover, the seroprevalence of *T. gondii* infection varied in different geographical areas with a general tendency of lower infection rate in the Northern region of Yangtze River (1.44%) and higher infection rate in the Southern region of Yangtze River (4.62%) (**Table 1**). However, the infection rates in Hainan, Sichuan and Chongqing are the highest (**Table 3**). The causes for these variations in infection rates in the areas are not yet known. Environmental conditions and regional climate may determine the degree of natural distribution of *T. gondii* infection. It has been reported that infection is more prevalent in warm climates and high rain fall areas than in cold climates and dry areas (Dubey, 2010). For the three provinces with the highest infection rates, the habit of food consumption may be the main reason. People in Hainan Province consume more sea food than other areas and instant-boiling meat is very popular in Chongqing and Sichuan areas. Further, environmental conditions favoring sporulation and survival of oocysts may be another reason for the high prevalence in these regions. Rainfalls in Southern China are much heavier than that in the North, which facilitate and oocysts survived outdoors in soil will be brought to the surface and spread to other places to expand the scope of *T. gondii* infection. In addition, it is likely that oocysts are easily carried into homes on shoes in rainy and humid conditions (Kniel et al., 2002; Dubey, 2010). Moreover, drying under low humidity in the North of the Yangtze River were deleterious to oocysts (Dubey, 2010).

In this study, drinking unboiled water is found to be a significant risk factor for *T. gondii* seropositivity among these participants (*p* = 0.028, adjusted OR = 3.1, CI: 1.9–5.2) (**Table 2**). This result indicated that contamination of drinking water by *T. gondii* are common, and drinking boiled water is a way of avoiding *T. gondii* infection in China. The result also speaks for the importance of the surveillance of water hygiene.

Among the participants in this study, both living in rural areas and gardening or agriculture are also significant risk factors for *T. gondii* infection, which was similar to the previous study (Mahmoudvand et al., 2015). The prevalence of *T. gondii* seropositivity was significantly higher in the participants living in rural areas (2.59%) than those living in cities (1.33%) (*p* = 0.009; adjusted OR = 0.5; 95% CI: 0.3–0.8) (**Table 2**). Individuals with frequent gardening and farming activities are more easily exposure to *T. gondii* (*p* = 0.000; adjusted OR = 3.1; 95% CI: 1.9–5.2) (**Table 2**), which were independent risk factors for *T. gondii* seropositivity by multivariate logistic regression analysis (*p* = 0.001; adjusted OR = 3.0; 95% CI: 1.5–5.7) (**Table 4**). These results indicated that people living in rural areas or farming had more chance to contact with oocysts shed in the feces of infected cats. The finding that *T. gondii* infection rate of ranging chicken was much higher than that of caged chicken also support our data (Xiao et al., 2010).

Moreover, we found that fresh oyster consumption was another potential risk factor for *T. gondii* infection though the difference was not statistically significant (**Tables 2, 4**), which was similar to the study from the United States (Jones et al., 2009). It was reported that *T. gondii* oocysts could be washed into the sea via runoff (Lindsay et al., 2003; Dubey, 2004), and oysters, clams, and mussels could ingest oocysts directly from seawater (Lindsay et al., 2003; Dubey, 2004, 2010). In China, fresh oyster has become more popular in recent years, this may explain the higher infection rate in the participants originated from Hainan Province.

In this study, the seroprevalence of *T. gondii* infection showed a general tendency with aging. Meanwhile, seroprevalence of *T. gondii* infection in postgraduate students (2.46%) (≥22-years-old) was higher than that in undergraduates (1.63%) (≤21-years-old). These results suggested that older participants may have more chance of *T. gondii* infection, though there was no statistical significance between the two groups. This is in agreement with other reports (Agmas et al., 2015; Cong et al., 2015). In addition, the anti-*T. gondii* IgG-seropositive rates was 1.91% (26/1,360) and 1.31% (29/2,209) in men and women (*p* = 0.751), respectively, contrary to the previous report (Zemene et al., 2012). This might be due to the living conditions which are very different between the countries.

Cats are the main definite host of *T. gondii*, and oocysts are shed from infected cat feces constantly, which are a major source of *T. gondii* infection in humans (Dubey, 2010). Contact with cats is consistently demonstrated to be a risk factor in prior seroepidemiologic studies (Chiang et al., 2012; Zemene et al., 2012; Agmas et al., 2015; Cong et al., 2015; Rodrigues et al., 2015). However, this study did not show any association between *T. gondii* infection with keeping cats (*p*>0.5). According to the questionnaire survey, although these students once had cats, they rarely contacted with the cats because of perennial learning in school.

## Conclusion

This study has shown that the general infection rate of *T. gondii* among the newly enrolled undergraduates and postgraduate students in China was 1.82% which is much lower than that obtained from the total population. Four variables were found to be associated with *T. gondii* infection, including geographical location, living in rural areas, gardening or agriculture, and drinking unboiled water by the univariate logistic regression, and only gardening or agriculture was the independent risk factor for *T. gondii* seropositivity by using multivariate logistic regression in this study.

## Author Contributions

NY and QC conceived and designed the study. NY, DW, MX, CL, JL, and AW performed the study. XS, YF, and NJ helped to collected the samples. NY and QC wrote the manuscript.

## Conflict of Interest Statement

The authors declare that the research was conducted in the absence of any commercial or financial relationships that could be construed as a potential conflict of interest.
